# Structure-Based Rational Design of a Toll-like Receptor 4 (TLR4) Decoy Receptor with High Binding Affinity for a Target Protein

**DOI:** 10.1371/journal.pone.0030929

**Published:** 2012-02-17

**Authors:** Jieun Han, Hyun Jung Kim, Sang-Chul Lee, Seungpyo Hong, Keunwan Park, Young Ho Jeon, Dongsup Kim, Hae-Kap Cheong, Hak-Sung Kim

**Affiliations:** 1 Department of Biological Sciences, Korea Advanced Institute of Science and Technology (KAIST), Yuseong-gu, Daejeon, Korea; 2 Division of Magnetic Resonance Research, Korea Basic Science Institute, Cheongwon, Chungbuk, Korea; 3 College of Pharmacy, Chungbuk National University, Cheongju, Chungbuk, Korea; 4 Department of Bio and Brain Engineering, Korea Advanced Institute of Science and Technology (KAIST), Yuseong-gu, Daejeon, Korea; 5 Graduate School of Nanoscience and Technology, Korea Advanced Institute of Science and Technology (KAIST), Yuseong-gu, Daejeon, Korea; University of Washington, United States of America

## Abstract

Repeat proteins are increasingly attracting much attention as alternative scaffolds to immunoglobulin antibodies due to their unique structural features. Nonetheless, engineering interaction interface and understanding molecular basis for affinity maturation of repeat proteins still remain a challenge. Here, we present a structure-based rational design of a repeat protein with high binding affinity for a target protein. As a model repeat protein, a Toll-like receptor4 (TLR4) decoy receptor composed of leucine-rich repeat (LRR) modules was used, and its interaction interface was rationally engineered to increase the binding affinity for myeloid differentiation protein 2 (MD2). Based on the complex crystal structure of the decoy receptor with MD2, we first designed single amino acid substitutions in the decoy receptor, and obtained three variants showing a binding affinity (K_D_) one-order of magnitude higher than the wild-type decoy receptor. The interacting modes and contributions of individual residues were elucidated by analyzing the crystal structures of the single variants. To further increase the binding affinity, single positive mutations were combined, and two double mutants were shown to have about 3000- and 565-fold higher binding affinities than the wild-type decoy receptor. Molecular dynamics simulations and energetic analysis indicate that an additive effect by two mutations occurring at nearby modules was the major contributor to the remarkable increase in the binding affinities.

## Introduction

Repeat proteins, which are composed of varying numbers of repeating modules (units), have been identified in a variety of functionally related proteins, and their modular architecture was shown to evolve to be suitable for protein-protein interactions [1], [2], [3]. Leucine-rich repeat (LRR) proteins, a typical repeat protein, are characterized by an assembly of consecutive LRR modules that represent the β strand-turn-α helix structure [4], [5], [6]. Repeat modules stack in a relatively linear manner to form an elongated modular architecture that has a horseshoe shape, providing large interaction surfaces. LRR proteins mediate many important biological functions including cell adhesion, signaling processes, neural development, bacterial pathogenicity, extracellular matrix assembly, and immune response [7], [8]. Typical LRR proteins include the mammalian ribonuclease inhibitor [9], Toll-like receptors (TLRs) [10], bacterial internalins [11], [12], plant disease-resistance R proteins, and variable lymphocyte receptors (VLRs) from jawless fish. With unique structural features, repeat proteins have increasingly attracted much attention as alternative scaffolds to immunoglobulin antibodies to generate molecular binders for use in biotechnology and biomedical fields [13]–[18].

Toll-like receptor 4 (TLR4), a superfamily of LRR proteins, is the principal receptor mediating innate immune responses against infections by Gram-negative bacteria [19], [20], [21]. In this process, myeloid differentiation protein 2 (MD2) recognizes various lipopolysaccharides (LPSs) released from the Gram-negative bacteria, forming a complex with LPS at its hydrophobic pocket [22]. The resulting MD2/LPS complex triggers a TLR4-mediated signaling process by binding to the TLR4 extracellular domain, leading to nuclear factor-kB activation, which causes acute and severe inflammation and sepsis. In an attempt to prevent the systemic immune response caused by LPS, a TLR4 decoy receptor was constructed by combining LRR modules from the TLR4 ectodomain and VLR [23]. The constructed decoy receptor was shown to attenuate the TLR4-mediated signaling process by trapping MD2, implicating a potential therapeutics for bacteria-induced sepsis and inflammation [24].

Design of a binding protein with high affinity and specificity for a target ligand is prerequisite for the use in biotechnology and biomedical fields, and many advances have been made [25], [26]. Nonetheless, engineering interaction interfaces and understanding molecular basis for affinity maturation of repeat proteins still remains a challenge. Here, we present a structure-based rational design of a repeat protein with high binding affinity for a target protein. A TLR4 decoy receptor composed of hybrid LRR modules was used as a model repeat protein, and its interaction interface was rationally designed to increase the binding affinity for MD2. Our strategy for increasing the binding affinity was to strengthen the pre-existing interactions and to generate additional intermolecular interactions with the target protein. Based on the complex crystal structure of the decoy receptor with MD2, we first designed single amino acid substitutions in the decoy receptor that would lead to increased binding affinity for MD2. We selected three of the variants showing a binding affinity (K_D_) one-order of magnitude higher than the wild-type decoy receptor, and determined their crystal structures to elucidate the interacting modes and contributions of individual residues. To further increase the binding affinity, single positive mutations were combined, and two double mutants were shown to have approximately 3000- and 565-fold higher binding affinities than the wild-type decoy receptor. To understand the molecular basis for a remarkable increase in the binding affinity, molecular dynamics simulations and energetic analysis were conducted.

## Results

### Rational design of single variants with increased binding affinity

**Figure 1:**
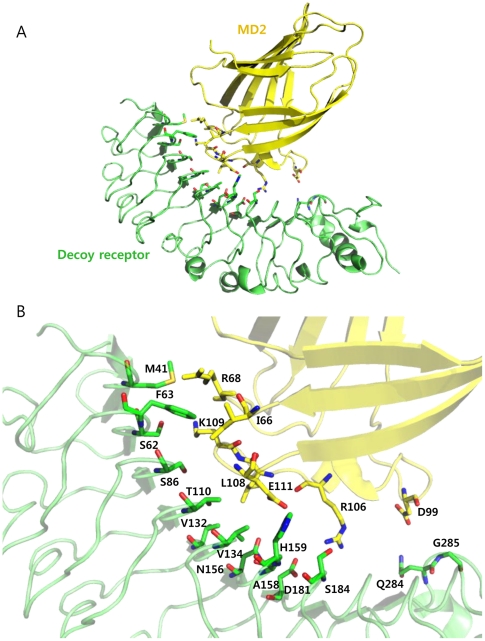
Identification of the mutation sites on the decoy receptor, TV3, for constructing single variants. (A) Crystal structure of the decoy receptor in complex with MD2 (PDB ID 2Z65). The decoy receptor and MD2 are shown as green and yellow, respectively. (B) Structure of the interaction interface. 14 identified residues in TV3 are indicated in green, and potential interaction residues in MD2 are represented in yellow.

To demonstrate a rational design of a repeat protein with high binding affinity, we employed a TLR4 decoy receptor as a repeat protein scaffold. This TLR4 decoy receptor was constructed by combining LRR modules from human TLR4 ectodomain and hagfish variable lymphocyte receptor (VLR) [23]. Overall structure of the decoy receptor comprised an LRRNT, seven LRR human TLR4 modules, and two LRRs, LRRCT of hagfish VLRB. 61. Based on the crystal structure of the decoy receptor in complex with MD2 (PDB ID 2Z65), we first attempted to identify the interacting residues and potential interacting residues on the decoy receptor that would increase the binding affinity toward MD2. For this, we examined the residues at the interface, which are located within 6 Å distance from the MD2, and selected the 14 residues located at eight repeat modules. These residues were expected to interact with Ile-66, Arg-68, Asp-99, Arg-106, Leu-108, Lys-109, and Glu-111 on MD2 (**Figure 1**). We adopted two approaches to design the decoy receptor variants with higher binding affinity for MD2: one was to strengthen the pre-existing interactions, and the other was to generate additional interactions by mutating the potential interaction residues based on the following analysis.

#### Hydrophobic interactions

By considering the size and hydrophobic index of each amino acid residue, we determined the substituting residues leading to increased hydrophobic interaction. Changing phenylalanine at position 63 was likely to form a hydrophobic interaction with Ile-66 of MD2 in the crystal structure of the decoy receptor/MD2 complex. Phe-63 was substituted for tryptophan and leucine for the interaction with Ile-66 in MD2. Analysis of the interaction interface inferred that four residues (Thr-110, Val-132, Val-134, and Asn-156) on the decoy receptor were in position to make additional hydrophobic interactions with Leu-108 of MD2 when substituted for larger amino acid residues. Replacement of these four residues with more hydrophobic residues was expected to generate additional hydrophobic interactions with Leu-108 on MD2, consequently resulting in an increase in MD2 binding affinity. Specifically, replacing Thr-110 with more hydrophobic residues such as isoleucine, leucine, phenylalanine, or tyrosine would generate additional interactions. In the case of Val-132 and Val-134, replacing these residues with phenylalanine or leucine might create a strong hydrophobic interaction. Substituting Asn-156 for isoleucine or phenylalanine appeared to produce an additional hydrophobic interaction. To minimize the number of variants to construct, the amino acid residue at each site was replaced with one or two representative residues, except Thr-110.

#### Charge interactions

Analysis of the decoy receptor/MD2 complex structure indicated that the residues at positions 41, 62, 86, 158, 184, 284, and 285 on the decoy receptor were located closely to the MD2 charged residues such as Arg-68, Asp-99, Lys-109, and Glu-111. We reasoned that substituting these residues for charged residues would increase the interaction with MD2 via charge interactions. It is likely that the M41E and S62E mutations would induce strong interactions with positively charged Arg-68 and Lys-109 of MD2. Additionally, replacing Ser-86 and Ala-158 with a negatively charged amino acid such as aspartic acid and a positively charged residue such as lysine, respectively, would generate additional charge interactions with Lys-109 and Glu-111 of MD2. Changing Ser-184 to glutamate or lysine was expected to form a strong interaction with Arg-106 and Glu-111 on MD2. Finally, mutating Gln-284 and Gly-285 to lysine would yield a charge interaction with Asp-99 of MD2.

#### Hydrogen bonds

From the crystal structure of the decoy receptor/MD2 complex, Ser-183 and Asp-209 on the decoy receptor had a hydrogen bond with Arg-106 of MD2. Furthermore, Asp-181 of the decoy receptor was located within a 5 Å from the Arg-106 of MD2, possibly producing an additional hydrogen bond. Replacing Asp-181 with a residue containing a long side chain such as glutamate would induce a hydrogen bond. Changing Val-134 and His-159 to residues with polar side chains such as asparagine and glutamine was predicted to yield additional hydrogen bonding with Glu-111 of MD2. Changing Ala-158 to phenylalanine appeared to strengthen a hydrogen bond between His-159 of decoy receptor and Glu-111 on MD2 by filling the vacant space between the two residues.

### Construction of single variants

Based on the decoy receptor designs described above, we constructed 22 single mutants by replacing the residues at 14 sites on the decoy receptor with one or two residues (**Table S1**). We first checked the expression of each variant, and observed that the expression levels of variants generally decreased compared to the wild-type decoy receptor. Nine of 22 single variants showed relatively high expression levels, including M41E, F63L, F63W, V132F, V134L, N156I, H159Q, D181E, and S184K. Dissociation constants (K_D_) of the selected variants for MD2 were determined using surface plasmon resonance (SPR) analysis. Typical sensograms of the wild-type decoy receptor and its variants are shown in **Figure S1**, and their K_D_ values are listed in **Table 1**. Of nine single mutants tested, eight exhibited increased affinity, and one had slightly decreased affinity for MD2. Interestingly, five single mutants (M41E, F63W, V134L, N156I, and H159Q) displayed binding affinities one-order of magnitude higher than that of the wild-type decoy receptor. This result strongly implied that our design strategy worked well and was reasonable.

**Table 1:** Binding affinities of single mutants for MD2.

Mutant	K_a_ (M^−1^ S^−1^)	K_d_ (S^−1^)	K_D_ (M)	Fold-increase
Wild-type	2.84±0.02×10^4^	2.22±0.05×10^−3^	7.80±0.14×10^−8^	1
M41E	1.64±0.57×10^4^	5.29±0.80×10^−5^	3.21±1.02×10^−9^	24.3
F63L	2.65±0.02×10^4^	1.15±0.01×10^−3^	4.33±0.03×10^−8^	1.8
F63W	1.39±0.10×10^4^	3.97±2.13×10^−5^	2.84±1.66×10^−9^	27.5
V132F	6.91±3.69×10^4^	1.54±0.01×10^−3^	2.22±1.41×10^−8^	3.5
V134L	2.67±0.28×10^4^	1.10±0.01×10^−4^	4.11±0.50×10^−9^	19.0
N156I	1.97±0.33×10^4^	8.05±4.77×10^−5^	4.07±1.71×10^−9^	19.2
H159Q	2.14±1.21×10^3^	1.00±0.44×10^−5^	4.30±3.23×10^−9^	18.1
D181E	1.91±0.10×10^4^	9.24±1.35×10^−4^	4.82±0.44×10^−8^	1.6
S184K	7.66±0.02×10^3^	1.41±0.02×10^−3^	1.83±0.04×10^−7^	0.4

Binding affinities (K_D_) of the single mutants for MD2 were measured from the association rate constants (K_a_) and dissociation rate constants (K_d_) using surface plasmon resonance. Fold-increase represents the ratio of binding affinities between the mutants and wild-type decoy receptor.

### Interface structures of the single variants

**Figure 2:**
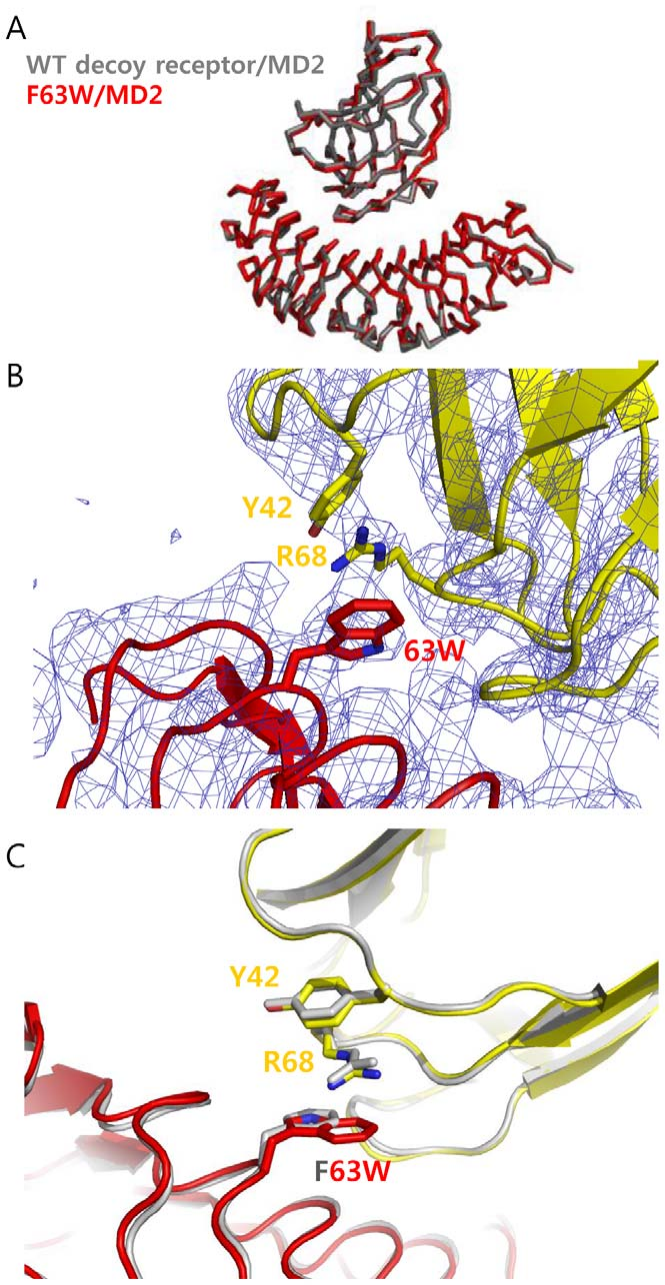
Crystal structure of the F63W mutant in complex with MD2. (A) Superimposed backbone structure of F63W mutant/MD2 complex into the wild-type decoy receptor/MD2 complex structure. The F63W showed a slight movement toward the N-terminal direction by 0.4 Å compared to the wild-type decoy receptor in complex with MD2. (B) The complex structure of F63W mutant/MD2. The mutated Tyr-63 was closely located to Arg-68 on MD2, creating cation-π interaction. The F63W mutant in complex structure is indicated as red and MD2 of F63W/MD2 complex is colored in yellow. The electron density map of mutant complex is shown as blue. (C) Comparison of the wild-type decoy receptor/MD2 and F63W/MD2 complex structures. The key residues and backbone structure of wild-type decoy receptor/MD2 complex are shown in grey. In F63W/MD2 complex, the F63W mutant is shown in red, and MD2 in yellow, respectively.

To get detailed information on interacting interfaces, we attempted to determine the crystal structures of three single variants (M41E, F63W, V134L) in complex with MD2 in the presence of Eritoran. It was shown that Eritoran, derived from the lipid A structure of *Rhodobacter sphaeroides* LPS, is critical for crystallizing the decoy receptor/MD2 complex [23]. We obtained the crystals of three mutants in complex with MD2 and Eritoran. The F63W mutant /MD2 complex structure was determined at a 3.6 Å resolution and the others at lower resolution. Even at this moderately low resolution, the density maps clearly reveal backbone structure and some side chains. Especially, we could determine the side chain position of the mutated F63W residue and interaction with Arg-68 of MD2. Superposition of the crystal structure of the mutants in complex with MD2 and Eritoran on the crystal structure of the wild-type decoy receptor/MD2/Eritoran complex resulted in a C_α_ r.m.s.d of 0.45 Å for all atoms (**Figure 2A**). MD2 bound to the concave surface derived from the “LxLxxN” parts of the LRR modules in the mutants. Eritoran bound to MD2 in a similar manner to the wild-type decoy receptor/MD2/Eritoran complex. From the complex structure of F63W mutant, we confirmed that the mutated Trp-63 was positioned to interact directly with Arg-68 of MD2 and the guanido group of Arg-68 on MD2 was placed between the indole ring of Trp-63 on the decoy receptor and the hydroxylphenyl group of Tyr-42 on MD2, creating an amino-aromatic (cation-π) interaction (**Figure 2B**). The larger Trp-63 indole ring was able to be properly positioned to form a cation-π interaction, whereas the Phe-63 phenyl ring was too far from Arg-68 in the wild-type decoy receptor/MD2 complex structure.

**Figure 3:**
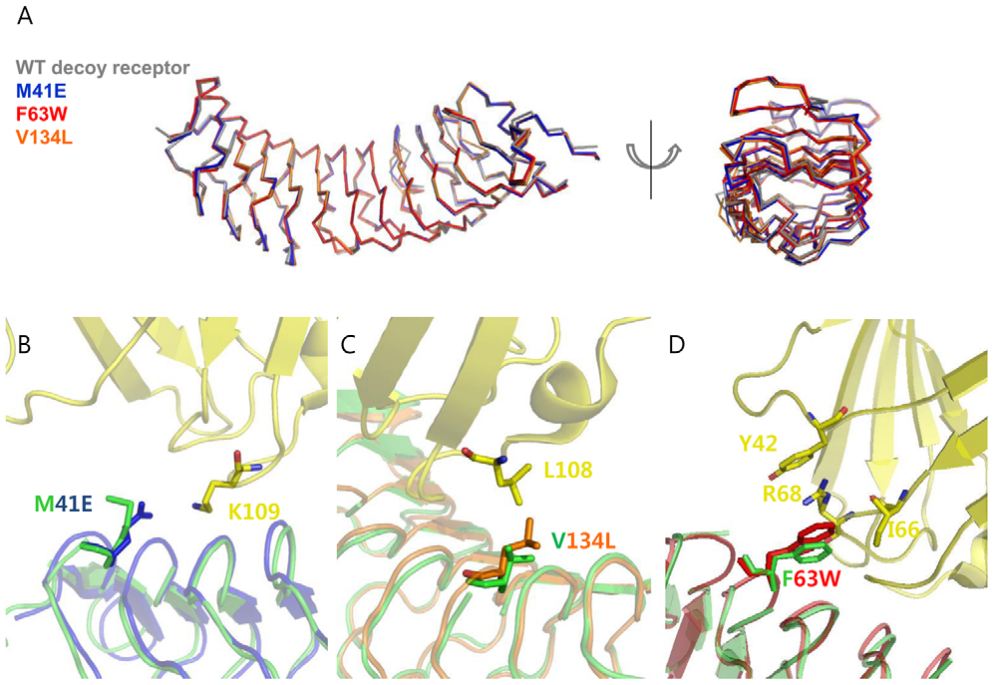
Interface structures of single variants. (A) Superimposed backbone structures of the wild-type decoy receptor and three single mutants (M41E, F63W, and V134L). (B) Interface structure of the M41E mutant obtained by superimposing the crystal structure of apo M41E mutant into the wild-type decoy receptor/MD2 complex structure. Glu-41 was positioned to be able to form a salt-bridge with Lys-109 on MD2. The crystal structure of the M41E is shown in blue, and the wild-type decoy receptor and MD2 are colored in green and yellow, respectively. (C) Interface structure of the V134L mutant obtained by superimposing the crystal structure of apo V134L mutant into the wild-type decoy receptor/MD2 complex structure. The larger Leu-134 is possible to make closer contact with Leu-108 on MD2. The crystal structure of the V134L is colored in orange (D) Interface structure of the F63W mutant obtained by superimposing the crystal structure of apo F63W mutant into the wild-type decoy receptor/MD2 complex structure. The mutated Trp-63 was expected to create the amino-aromatic (cation-π) interaction of Tyr-42 and Arg-68. The crystal structure of the F63W is shown in red.

To analyze the interaction interfaces of other single mutants (M41E and V134L), we obtained the modeled interaction interfaces by superimposing their apo structures into the complex structure of the wild-type decoy receptor/MD2. For this, we determined the crystal structures of the single mutants in free form at a −2.4 Å resolution. As shown in **Figure 3A**, the single mutants were well superimposed on the wild-type decoy receptor, showing a C_α_ r.m.s.d of 0.4–0.5 Å, which indicates that their original backbone structures were retained. The relative C_β_ positions of the mutated residues were also well superposed on those of the wild-type. The concave surface of the mutants composed of seven parallel β strands remained unchanged as a whole compared to the wild-type. In the M41E mutant, a substituted glutamate residue was expected to interact with Lys-109 of MD2, creating a salt-bridge (**Figure 3B**), whereas Met-41 in the wild-type decoy receptor had no specific interaction with MD2. This salt bridge is likely to contribute to an increase in binding affinity of the M41E mutant for MD2 by changing the electrostatic property. In the V134L mutant, a substituted Leu-134 residue was able to form a hydrophobic interaction with Leu-108 on MD2 (**Figure 3C**). It seemed that the larger side chain of Leu-134 had closer contact with Leu-108 on MD2 without a significant change in the backbone structure compared with the wild-type decoy receptor in complex with MD2. To validate the modeled interaction interfaces of the single mutants (M41E and V134L), we also obtained the modeled interaction interface of the F63W mutant with MD2 by superimposing the crystal structure of apo F63W mutant into the wild-type decoy receptor/MD2 complex as the M41E and V134L mutants. As can be seen **Figure 3D**, the modeled interface structure of the F63W mutant was well coincident with its crystal structure in complex with MD2. This result strongly supports that the modeled interaction interfaces of the M41E and V134L mutants with MD2 are valid.

### Construction and analysis of multiple mutants

Consecutive modules in repeat proteins are closely associated with each other to form an elongated unique structure, providing a large surface area for interaction with a target ligand. We reasoned that multiple mutations over repeat modules of the decoy receptor composed of eight LRR units would lead to a significant increase in MD2 binding affinity. Based on the results of single mutants, 13 multiple mutants were constructed by combining single mutations; 12 double mutants and one triple mutant (**Table S1**). Of them, we selected five double mutants showing a relatively high expression level, and determined their binding affinities for MD2. As shown in **Table 2**, two double mutants, M41E/F63W and V134L/H159Q, displayed remarkably increased binding affinities for MD2. Their K_D_ values were estimated to be 26 pM and 138 pM, which corresponded to approximately 3000- and 565-fold increases compared to the wild-type (K_D_ = 78 nM), respectively (**Figure S1**). Meanwhile, the F63W/D181E mutant exhibited a comparable affinity (K_D_ = 39 nM) to the wild-type. Analysis of the association and dissociation rate constants of the M41E/F63W and V134L/H159Q mutants revealed that their association rate constants (K_a_) were almost comparable to the wild-type decoy receptor. In contrast, their dissociation rate constants (K_d_) significantly decreased, which resulted in a remarkable increase in the binding affinities of the mutants for MD2. This result strongly supports the presumption that an appropriate combination of single mutations over individual modules of repeat proteins would induce an additive effect on binding affinity. In particular, it is noteworthy that mutations occurring at nearby modules resulted in a notable increase in the interaction between the mutants and MD2.

**Table 2:** Binding affinities of double mutants for MD2.

Mutant	K_a_ (M^−1^ S^−1^)	K_d_ (S^−1^)	K_D_ (M)	Fold- increase
Wild-type	2.84±0.02×10^4^	2.22±0.05×10^−3^	7.80±0.14×10^−8^	1
M41E/F63W	2.01±0.01×10^4^	5.23±0.5×10^−7^	2.60±0.24×10^−11^	3000
M41E/H159Q	3.16±2.60×10^3^	4.61±3.90×10^−4^	1.45±0.48×10^−7^	0.5
F63W/V134L	3.66±0.01×10^3^	1.03±0.22×10^−3^	2.83±0.62×10^−7^	0.3
F63W/D181E	5.83±0.32×10^2^	2.27±0.92×10^−5^	3.90±1.80×10^−8^	2
V134L/H159Q	4.41±0.60×10^4^	6.12±1.11×10^−6^	1.38±0.06×10^−10^	565

Fold-increase indicates the ratio of binding affinities between the mutants and wild-type decoy receptor.

To gain some insight into the mechanism by which the binding affinities of double mutants (M41E/F63W and V134L/H159Q) significantly increased, we conducted molecular dynamics simulations for the model structures of the double mutants in complex with MD2 to analyze the interaction strengths and binding modes. We attempted to determine the crystal structures of these mutants in complex with MD2, but could not obtain the appropriate complex crystals for a structural determination. Thus, we obtained the model structures of the double mutants and used them for molecular dynamics simulation. Only mutated residues were subjected to binding energy calculations, because the energy fluctuation in the system was too large to converge in a 5-ns time scale. Based on the simulation results, we estimated the Coulomb interactions and Lennard-Jones (LJ) potentials as well as the number of hydrogen bonds between each double mutant and MD2. Differences in the energy levels between respective mutants and wild-type decoy receptor in complex with MD2 are summarized in **Table 3**. For comparison, we performed the same calculations for the single mutant crystal structures.

**Table 3:** Changes in the interaction energies and hydrogen bond numbers.

Mutants	ΔCoulomb*(kJ/mol)	ΔLJ*(kJ/mol)	ΔHB†
M41E	−86.32 (−48.14)	4.53 (5.14)	1.27 (0.63)
F63W	−17.08 (3.82)	−11.02 (−2.08)	0.80 (0.04)
V134L	−1.49 (−1.38)	−4.97 (−1.80)	0 (0)
H159Q	−26.10	3.93	0.35
D181E	−9.00	0.60	0.38
M41E/F63W	−150.41	0.30	2.53
F63W/D181E	21.79	4.68	−0.01
V134L/H159Q	−32.28	−1.75	0.50

*Differences in the interaction energies between the mutants and wild-type decoy receptor (E_mut_-E_wild_, kJ/mol) were calculated using Gromacs4 package with an Amber03 force field. Each energy value represents the average of 3-ns molecular dynamics trajectories.

† The number of hydrogen bonds was analyzed for each 3-ns molecular dynamics trajectory snapshot, and differences in their average values between the mutants and wild-type decoy receptor are shown as ΔHB.

Values in parenthesis were obtained by molecular dynamics simulations with the crystal structures of the single mutants (M41E, F63W, and V134L).

In the M41E/F63W double mutant showing a 3000-fold increased binding affinity, the change in the Coulomb interaction was dominant, as it increased by 150.41 kJ/mol, whereas a negligible change (0.3 kJ/mol) in the LJ potential was observed compared with the wild-type. In addition, the number of hydrogen bonds increased by 2.53. However, the M41E mutant exhibited an increase of 86.31 kJ/mol in the Coulomb interaction and a decrease of the LJ potential by 4.53 kJ/mol, respectively. Thus, an increase in the binding affinity of the M41E mutant was likely to come mainly from the increased charge interaction. The F63W mutant displayed increases in the Coulomb interaction and LJ potential by 17.08 and 11.02 kJ/mol, respectively, indicating a significant enhancement in the hydrophobic interaction, as expected. Trp-63 concurrently contributed to an increase in the charge interaction via additional hydrogen bonding with the residues on MD2. Increases in the Coulomb interaction in the double mutant (M41E/F63W) were much higher than those obtained by summation of two single mutations, indicating that two single mutations (M41E and F63W) are additive in an interaction with MD2, resulting in a remarkable increase in binding affinity.

As for the V134L/H159Q double mutant, the Coulomb interaction and LJ potential were stabilized by −4.69 kJ/mol and −0.71 kJ/mol, respectively, compared with those obtained by adding two single mutations (V134L and H159Q). At the same time, H159Q produced an increased number of hydrogen bonds in the double mutant from 0.35 to 0.5, compared with the wild-type decoy receptor. Although the increase in the Coulomb interaction was relatively low, this mutant displayed a 565-fold increase in binding affinity. Other factors contributing to the increased binding affinity remain to be demonstrated. Unlike the above two double mutants, combining F63W and D181E resulted in a marginal increase in MD2 binding affinity, which seemed to originate from the destabilized Coulomb interaction of 21.79 kJ/mol and the destabilized LJ potential of 4.68 kJ/mol. Similar results were observed in the MD simulations of the single mutant crystal structures, which verifies that our simulations are reasonable.

**Figure 4:**
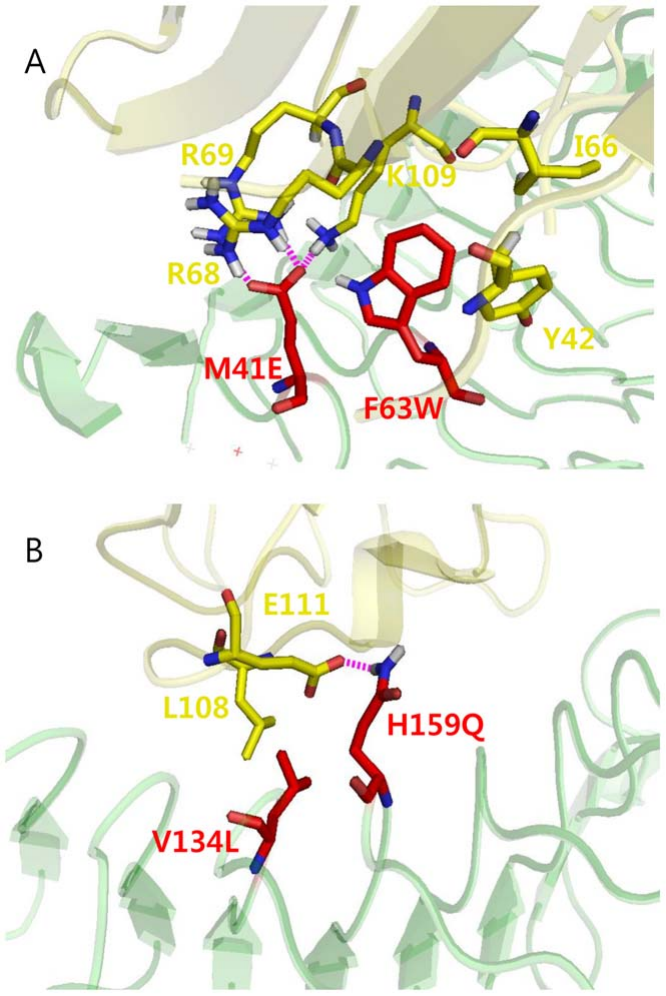
Snapshots of molecular dynamics simulation trajectories. Each snapshot was chosen to represent the changes in interaction energies. (A) Modeled structure of the double mutant, M41E/F63W. Increased hydrophobic environment resulting from the F63W mutation induced strong charge interactions between Glu-41 of the decoy receptor and Arg-68, Arg-69, and Lys-109 on MD2. (B) Modeled structure of the double mutant, V134L/H159Q. The V134L mutation led to increased hydrophobic interaction, strengthening the charge interaction between Gln-159 on decoy receptor and Glu-111 on MD2.

To obtain more detailed information about the additive effect by two mutations on the binding affinity of the double mutant (M41E/F63W) for MD2, we analyzed the effect of the F63W mutation on the interaction of Glu-41 with water molecules in the double mutant. The Coulomb interaction between Glu-41 and water molecules in the double mutant was calculated and compared with that of the M41E mutant. The energy levels represented the changes from the wild-type. The Coulomb interaction in the single mutant was approximately −325.09 kJ/mol, whereas the double mutant had a Coulomb interaction of −190.80 kJ/mol. A significant decrease in the Coulomb interaction in the double mutant reflected an increase in the interaction of the mutant with MD2, which was caused by Trp-63. In other words, replacement with a more hydrophobic residue (tryptophan) at position 63 enhanced hydrophobicity, leading to exclusion of water molecules, which consequently resulted in the reduced Coulomb interaction with water molecules. At the same time, the F63W mutation resulted in a subtle change in conformation, enabling Glu-41 to form multiple interactions with the residues on MD2. As shown in **Figure 4A**, Glu-41 in the double mutant formed multiple interactions with Arg-68, Arg-69, and Lys-109 of MD2. The structural analysis also revealed that the double mutant (M41E/F63W) underwent a slight change in conformation compared with the single M41E mutant. In this context, it is evident that two single mutations (M41E and F63W) were additive, yielding a 3000-fold increase in the binding affinity for MD2. In the V134L/H159Q mutant, an increased hydrophobic environment, resulting from V134L, enhanced the charge interactions between Gln-159 and Glu-111 of MD2, reducing the interactions with water molecules (**Figure 4B**). In contrast, in the case of the F63W/D181E mutant, two single mutations were located far apart; thus, the local hydrophobic effect from mutated Trp-63 may have been very weak, resulting in a negligible effect on the charge interaction by Glu-181. Additionally, this might hinder the interaction between the mutated Glu-181 and Arg-106 of MD2, leading to a reduction in the number of hydrogen bonds, ΔHB = −1.18.

## Discussion

We have demonstrated a structure-based rational design of the TLR4 decoy receptor with high binding affinity for MD2. Design of single substitutions based on the interaction interface generated the single variants showing a considerable increase in binding affinities compared with the wild-type decoy receptor. The crystal structures of three single mutants revealed that mutated residues have interactions with the target protein (MD2) as we designed, which confirms that our design strategy works well. A significant increase in the binding affinity was achieved by combining single positive mutations, and double mutants (M41E/F63W and V134L/H159Q) were shown to have about 3000- and 565-fold increased binding affinities for the target, respectively. Molecular dynamics simulations and energetic analysis of the double variants indicated that an additive effect by two mutations occurring at nearby modules plays a major role in a remarkable increase in the binding affinity. A strong additive effect seems to result from modular architecture of a repeat protein.

The crystal structures of the selected single mutants as apo and complexed with MD2 provided detailed information on the interaction modes of the residues at the interface and the contribution of mutated residues to binding affinity. Superposition of the mutants on the wild-type revealed that their original backbone structures were retained, which represents the structural characteristics of repeat proteins. The M41E mutant was designed to have a strong charge interaction with a positively charged Lys-109 in MD2 and the substituting Glu-41 was shown to be able to form an additional interaction with Lys-109 of MD2, resulting in a significant increase in binding affinity. From the structure of the F63W and V134L mutants, it was expected that hydrophobic interactions would increase by introducing a residue with a larger hydrophobic side chain. The complex structure of the F63W mutant displayed that Trp-63 was closely positioned to form a cation-π interaction with Arg-68 on MD2, confirming our design strategy. In the case of the V134L mutant, the larger Leu-134 was able to have a strong hydrophobic interaction with Leu-108 on MD2, which seemed to have a positive effect on binding affinity. This result also validates our strategy for the design of variants with high binding affinities for MD2. Molecular dynamics simulations supported the above results as shown in **Table 3**. In the M41E and D181E mutants, substitutions strengthened the Coulomb interactions. Both the F63W and V134L mutations were designed to enhance hydrophobic interactions and MD simulations confirmed increased LJ potentials. Interestingly, these mutations were predicted to enhance the charge interactions as well. The H159Q mutant was expected to have additional hydrogen bonds with MD2 compared with the wild-type decoy receptor. These results are coincident with the recent reports that electrostatic interactions are significant in TLR4/MD2/LPS signaling as well as in antagonist design related to TLR4 signaling pathway [27], [28].

Combining positive single mutations for further increasing the binding affinity generated the double mutants (M41E/F63W and V134L/H159Q) with about 3000- and 565-fold increased binding affinities for the target, respectively. Interestingly, two mutations occurred at nearby modules, and these mutations were shown to be additive for increasing an interaction with the target. In other words, Trp-63 enhanced the hydrophobic interaction, and this mutation concurrently led to a significant increase in the Glu-41 charge interaction. In the simulation model, the mutated Glu-41 made a charge interaction with three residues (Arg-68, Arg-69, and Lys-109 of MD2 (**Figure 4A**). Increasing the hydrophobic interaction would create a more hydrophobic local environment, excluding water molecules, which consequently would result in much stronger Coulomb interactions between the mutant and the target. Molecular dynamics simulations of the single and double mutants also provided a detailed explanation on an additive effect by two single mutations occurring at nearby modules. Interactions between mutated polar residues and water molecules were predicted to be significantly weakened by Trp-63. The V134L/H159Q mutant also had a similar effect to the M41E mutant, even though the change in the interaction with water molecules was not as high as that of the M41E/F63W mutant. Thus, the closely localized mutations effectively strengthened the hydrophobic and charge interactions, inducing a significant additive effect.

In conclusion, we have demonstrated a successful design of the TLR4 decoy receptor with high binding affinity for MD2 by a rational approach. An additive effect by two mutations occurring at nearby modules was shown to be a major contributor to the remarkable increase in the binding affinity for the target. With unique structural features such as modular architecture and rigid backbone structure, repeat proteins have increasingly attracted much attention as alternative scaffolds. In addition, they evolved to be suitable for protein-protein interactions, mediating many important biological functions in vivo. The present study will provide some insights into designing repeat proteins with high binding affinity and specificity as well as understanding the molecular basis for protein-protein interactions.

## Materials and Methods

### Gene cloning and mutant construction

The gene encoding the TLR4 decoy receptor, TV3, was cloned into the pAcGP67 vector (BD Biosciences) with the BamH I and Not I sites. For protein purification, a thrombin cleavage site and the Fc domain of human IgG were cloned into Not I and Bgl II of the pAcGP67 vector containing the decoy receptor gene. The human MD2 was cloned as the fused form with Protein A (GE healthcare Life Sciences) into pAcGP67 vector using the same restriction enzyme sites. For the removal of Protein A tag, the thrombin cleavage site was introduced. The proper restriction enzymes (Takara) and DH5α (RBC) competent cells were used to clone the genes. Twenty-two decoy receptor variants with a single substitution were constructed from the wild-type by site-directed mutagenesis using overlapping PCR with proper primers [29]. The double and triple mutants were constructed by adding the second and third mutations into single mutants. Each cell containing the constructed mutant was cultured at 200 rpm in LB media at 37°C overnight. After cell lysis, the DNA preparation step was performed using a MIDI-preparation kit (Axygen) to obtain a high DNA concentration (>1 mg/ml) for protein expression using a baculovirus system.

### Protein expression and purification

A baculovirus system with the recombinant transfer vector, pAcGP67 (BD Biosciences) and Sf9 cells (Invitrogen) was used for virus production and amplification of genes coding for wild-type decoy receptor and the constructed mutants. A high DNA concentration corresponding to the genes and linearized baculovirus DNA were co-transfected to the Sf9 cell. SF9 cells were used for the virus amplification step. Hi5 cells (Invitrogen) were infected with the harvested virus and cultured for 3 days at 28°C. Secreted proteins were purified from the culture media using a column packed with rProteinA Agarose 6FF (Peptron). Following a washing step, the protein was subjected to thrombin digestion at 4°C overnight to remove the Fc tag. Wild-type decoy receptor and its variants with no tag were collected from the flow-through of resin. The mutant proteins were further purified using Q ion-exchange and gel filtration chromatography (GE Healthcare Life Sciences). Human MD2 fused to a protein A tag was co-expressed with the wild-type decoy receptor or its mutants in Hi5 cells followed by purification using a IgG Sepharose (GE Healthcare Life Sciences) affinity chromatography. The protein A tag was removed by thrombin digestion, and the MD2 complexed with either wild-type decoy receptor or its mutants was further purified using SP ion-exchange and gel filtration chromatography (GE Healthcare Life Sciences). To remove an aggregated or multimeric form of MD2, gel filtration chromatography using Superdex 75 was carried out using 20 mM Tris-Cl buffer (200 mM NaCl, pH 8.0), and monomeric MD2 was used for further experiments. The purification steps were performed at 4°C.

### Surface plasmon resonance (SPR)

The binding affinities (K_D_) of the decoy receptor and its variants with MD2 were obtained using SPR spectroscopy (Biacore 3000 System, GE Healthcare). Wild-type decoy receptor or its variants were covalently immobilized onto a carboxymethyl dextran surface of a CM5 chip using a standard EDC/NHS coupling method with sodium acetate buffer (pH 3.5–4.5). The amount of protein immobilized on the chip surface ranged from 500 to 800 resonance units (RU). Serially diluted MD2 solutions with HBS-EP buffer (GE Healthcare) were injected into the flow-cell at 60 µL/min for 90 sec, followed by a 30 min dissociation phase, and the changes in RU were traced as a function of time. The MD2 concentration ranged from 4 nM to 2 µM. Sensorgrams were corrected with a blank reference and fit with Biacore Evaluation software. The K_D_ values were determined by fitting the data to a 1∶1 Langmuir binding model using Biacore Evaluation software.

### Crystallization and structure determination

To determine the crystal structures of the decoy receptor variants in complex with MD2, the complex crystals were obtained in the presence of Eritoran, as described previously [23]. Eritoran was a generous gift from Eisai (Andover, USA). Briefly, Eritoran was sonicated for 10 min followed by incubation with the preformed mutant/MD2 complex at 37°C for 3 hr. The molar ratio of Eritoran to the protein was maintained at 10∶1. The decoy receptor variant /MD2/Eritoran complex was purified using Superdex 200 gel filtration chromatography to remove unbound Eritoran and used for crystallization. Crystals of the resulting complexes were obtained by mixing 1 µL of protein solution and 1 µL of crystallization solution after 3 days at 23°C. The crystals of the three variants were grown in 0.1 M Bis-Tris (pH 5.5) containing 36% w/v polyethylene glycol 1000 and 0.2 M lithium sulfate. The decoy receptor mutant/MD2/Eritoran complexes were crystallized in 0.1 M sodium acetate (pH 4.5), 22% w/v polyethylene glycol 8000, and 0.2 M lithium sulfate. For data collection, ethylene glycol was included as a cryoprotectant in the complex crystallization condition at a final concentration of 25%. A diffraction data was collected using the synchrotron X-ray source at the 4A beam line of the Pohang Accelerator Laboratory (Pohang, Korea) and the BL-17A beam line of the Photon Factory (Tsukuba, Japan). The diffraction images were processed with the HKL2000 package and the MOSFLM/SCALA programs. Initial phases were calculated by molecular replacement using PHASER and refined with REFMAC 5.0 [30]. The crystal structures of the decoy receptor alone (PDB ID 2Z62) and the decoy receptor/MD2/Eritoran complex (PDB ID 2Z65) were used as the search model. The molecular model was fit using the COOT graphic program [31], and the final models were further refined using the Phenix and CNS programs. Crystallographic and refinement statistics are summarized in Table S2.

### Modeling and simulation of variants

Modeled mutant structures were generated by searching a rotamer library using FoldX BuildModel module [32], [33] based on the crystal structure of the wild-type decoy receptor/MD2/Eritoran complex (PDB ID 2Z65) in pH 7 solution at 298 K. The structures of five single variants (M41E, F63W, V134L, H159Q, and D181E) and three double mutants (M41E/F63W, F63W/D181E, and V134L/H159Q) were modeled. Because the conformation of the MD2 Arg-68 on the F63W mutant was quite different from that of the native structure, it was corrected to have the native conformation by adjusting the torsion angles of Arg-68 and Trp-63 in PyMol (PyMOL Molecular Graphics System, Version 1.2r3pre, Schrödinger, LLC). Then, the system was modeled with Amber03 force field [34] and simulated with the Gromacs4 package [35]. In addition to the modeled structures, the resolved crystal structures for the three mutants, M41E, F63W, and V134L, were also used in simulations. The system was solvated with TIP3P water molecules and neutralized by adding chloride ions. We adopted the 5000 steps steepest decent energy minimization process and subsequent 50 ps equilibration restraining the motion of heavy atoms for simulation. The system was gradually heated from 0 to 310 K during 500 ps, and was maintained at 310 K with Berendsen's thermostat for 4.5 ns. The last 3 ns trajectories were used for the analysis.

### Trajectory analysis

Coulomb energy and the LJ potential between the residues of mutants and MD2 were recalculated from the MD trajectory using the “*-rerun*” option in *mdrun* of the Gromacs4 package. The number of hydrogens for the same group was analyzed by the *g_hbond* program in the Gromacs4 package.

## Supporting Information

Figure S1 **Binding affinities of the wild-type decoy receptor and variants for MD2 by surface plasmon resonance (SPR) measurements.** (A)Wild-type decoy receptor (B) M41E (C) F63W (D) V134L (E) M41E/F63W (F) V134L/H159Q.
(TIF)

Table S1 **Constructed decoy receptor variants with single and multiple mutations.** Underlined variants represent those which were selected based on the relative expression levels.
(DOC)

Table S2 **Crystallographic statistics.**
(DOC)
