# Supplementary figures and images for: Structure-Based Rational Design of a Toll-like Receptor 4 (TLR4) Decoy Receptor with High Binding Affinity for a Target Protein
Source: PLoS One. 2012 Feb 17;7(2):e30929. doi: 10.1371/journal.pone.0030929 (PMC3281905; doi:10.1371/journal.pone.0030929)

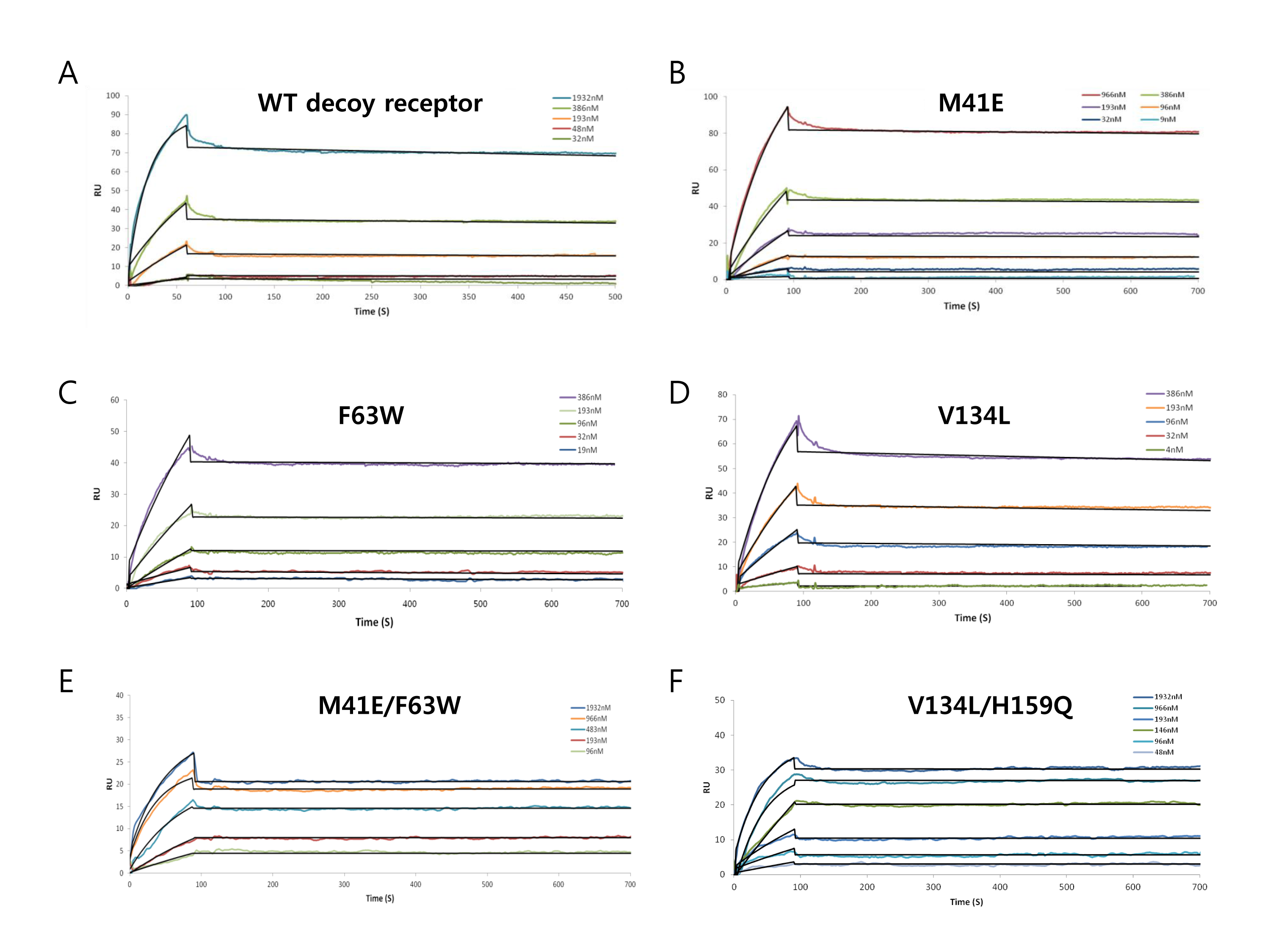

Supplement: Figure S1 — Binding affinities of the wild-type decoy receptor and variants for MD2 by surface plasmon resonance (SPR) measurements. (A)Wild-type decoy receptor (B) M41E (C) F63W (D) V134L (E) M41E/F63W (F) V134L/H159Q. (TIF) [file pone.0030929.s001.tif]
