# Supplementary material for: Structure-Based Rational Design of a Toll-like Receptor 4 (TLR4) Decoy Receptor with High Binding Affinity for a Target Protein
Source: PLoS One. 2012 Feb 17;7(2):e30929. doi: 10.1371/journal.pone.0030929 (PMC3281905; doi:10.1371/journal.pone.0030929)
Supplement: Table S1 — Constructed decoy receptor variants with single and multiple mutations. Underlined variants represent those which were selected based on the relative expression levels. (DOC) [file pone.0030929.s002.doc]

**Table S1.** Constructed decoy receptor variants with single and multiple mutations.

Underlined variants represent those which were selected based on the relative expression levels.

| 1 | M41E | 19 | S184K |
| --- | --- | --- | --- |
| 2 | S62E | 20 | S184E |
| 3 | F63L | 21 | Q284K |
| 4 | F63W | 22 | Q285K |
| 5 | S86D | 23 | M41E/F63W |
| 6 | T110 I | 24 | M41E/V134L |
| 7 | T110F | 25 | M41E/H159Q |
| 8 | T110L | 26 | M41E/N156I |
| 9 | T110Y | 27 | F63W/V132F |
| 10 | V132F | 28 | F63W/V134L |
| 11 | V134L | 29 | F63W/N156I |
| 12 | V134N | 30 | F63W/H159Q |
| 13 | N156I | 31 | F63W/D181E |
| 14 | N156F | 32 | V134L/N156I |
| 15 | A158F | 33 | V134L/H159Q |
| 16 | A158K | 34 | N156I/H159Q |
| 17 | H159Q | 35 | F63W/V134L/D181E |
| 18 | D181E |  | |
