# Supplementary material for: Structure-Based Rational Design of a Toll-like Receptor 4 (TLR4) Decoy Receptor with High Binding Affinity for a Target Protein
Source: PLoS One. 2012 Feb 17;7(2):e30929. doi: 10.1371/journal.pone.0030929 (PMC3281905; doi:10.1371/journal.pone.0030929)
Supplement: Table S2 — Crystallographic statistics. (DOC) [file pone.0030929.s003.doc]

**Table S2.** Crystallographic statistics.

|  | F63W/MD2/Eri | M41E | F63W | V134L |
| --- | --- | --- | --- | --- |
| Data collection |  | | | |
| Space group | P212121 | P212121 | P212121 | P212121 |
| Cell dimensions |  | | | |
| a, b, c (Å) | 80.2, 126.9, 129.5 | 32.0, 50.8, 191.9 | 32.8, 50.6, 191.4 | 32.0, 50.5, 192.8 |
| α, β, γ (°) | 90.0, 90.0, 90.0 | 90.0, 90.0, 90.0 | 90.0, 90.0, 90.0 | 90.0, 90.0, 90.0 |
| Resolution (Å) | 3.60 | 2.40 | 2.37 | 2.45 |
| Rsym or Rmerge | 0.126(0.356) | 0.084(0.423) | 0.077(0.256) | 0.081(0.382) |
| *I/σI* | 6.9(3.7) | 10.9(2.5) | 16.7(2.5) | 11.3(2.8) |
| Completeness (%) | 99.7(99.4) | 93.6(83.0) | 96.2(76.9) | 94.3(83.6) |
| Redundancy | 3.8 | 4.1 | 3.7 | 4.1 |
| Search Probes | TV3/MD2/Eri | TV3 | | |
| Refinement |  | | | |
| Resolution (Å) | 50-3.60 | 50-2.40 | 50-2.37 | 50-2.50 |
| No. reflections  (work/test) | 15021/769 | 10392/510 | 12402/669 | 10319/568 |
| Rwork/Rfree | 0.306 /0.341 | 0.238/0.306 | 0.231/0.271 | 0.240/0.300 |
| No. atoms |  | | | |
| Protein/water | 6652/- | 2187/38 | 2189/50 | 2196/32 |
| Sugars (CARB/HETE)/SO4 | (84/228) | (42/21)/15 | (42/21)/15 | (42/21)/5 |
| Average B factors |  | | | |
| Protein/Water | 87.34/- | 41.48/33.31 | 42.74/36.70 | 41.30/32.06 |
| Sugars (CARB/HETE)/SO4 | (100.00/92.86) | (83.30/97.15)/76.06 | (82.46/99.45)/69.13 | (72.76/88.00)/57.58 |
| R.m.s deviations |  | | | |
| Bond lengths (Å)/angles (°) | 0.010 /1.699 | 0.007/1.213 | 0.006/1.234 | 0.008/1.238 |
| Protein complexes in A. U. | 2 | 1 | 1 | 1 |

Highest resolution shell is shown in parenthesis. A. U., asymmetric units
